# Supplementary material for: Epigenetic and transcriptional dysregulation in CD4+ T cells in patients with atopic dermatitis
Source: PLoS Genet. 2022 May 16;18(5):e1009973. doi: 10.1371/journal.pgen.1009973 (PMC9135339; doi:10.1371/journal.pgen.1009973)

Supplemental Figure 1. ATAC-seq and NFKB1 ChIP-seq peak pairwise normalized read count comparisons. Each dot represents a peak that is present in either of the two samples. The X- and Y-axis indicate the number of normalized read counts for that peak in the two samples. The red line indicates peaks with similar normalized read counts between the two samples. **A**. ATAC-seq scatter plot analysis for controls. **B**. ATAC-seq scatter plot analysis for AD cases. **C**. ChIP-seq scatter plot analysis for controls. **D**. ChIP-seq scatter plot analysis for AD cases. E. Global Variability analysis of the ATAC and ChIP peaks across AD and controls.

A. ATAC-seq normalized read count pairwise comparison (controls)

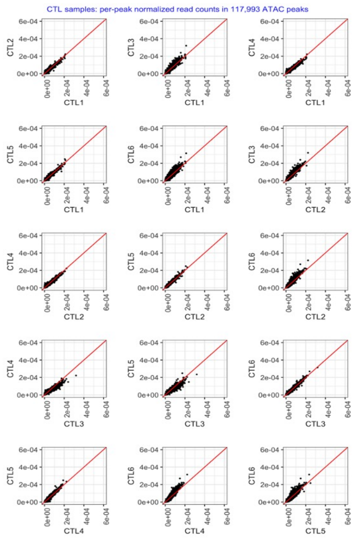

B. ATAC-seq normalized read count pairwise comparison (cases)

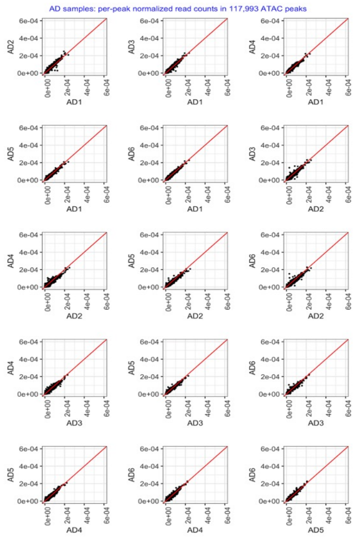

C. ChIP-seq normalized read count pairwise comparison (controls)

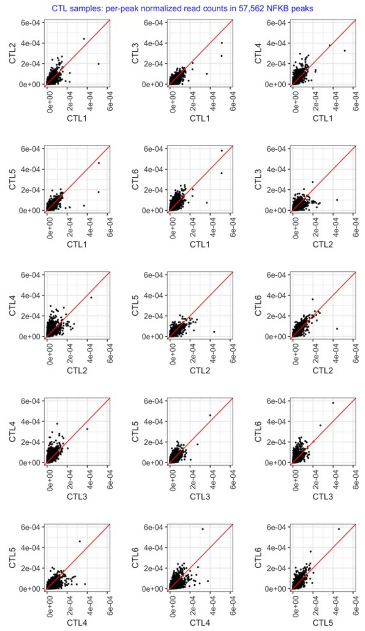

D. ChIP-seq normalized read count pairwise comparison (cases)

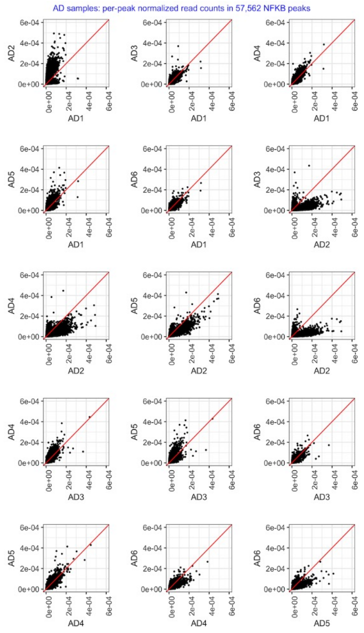

E. Global Variability analysis

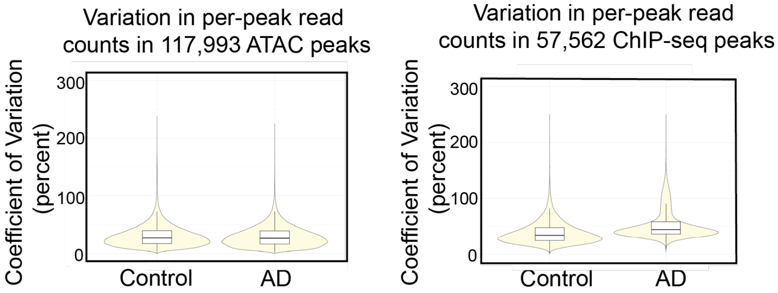

Supplement: S1 Fig — Each dot represents a peak that is present in either of the two samples. The X- and Y-axis indicate the number of normalized read counts for that peak in the two samples. The red line indicates peaks with similar normalized read counts between the two samples. A. ATAC- seq scatter plot analysis for controls. B. ATAC-seq scatter plot analysis for AD cases. C. ChIP-seq scatter plot analysis for controls. D. ChIP-seq scatter plot analysis for AD cases. E. Global Variability analysis of the ATAC and ChIP peaks across AD and controls. (PDF) [file pgen.1009973.s001.pdf]
