# Supplementary material for: Epigenetic and transcriptional dysregulation in CD4+ T cells in patients with atopic dermatitis
Source: PLoS Genet. 2022 May 16;18(5):e1009973. doi: 10.1371/journal.pgen.1009973 (PMC9135339; doi:10.1371/journal.pgen.1009973)

Supplemental Figure 3: Number of consistently differential ATAC-seq and ChIP-seq peaks across sample pairs. ATAC-seq and ChIP-seq peaks were called for all cases and controls. For each matched pair of samples, a MAnorm analysis identified shared peaks, control-specific peaks and AD-specific peaks (see Methods). Bar plots indicate the distributions of the number of control-specific (left) or case-specific peaks (right) for ATAC-seq (A,B) and ChIP-seq (C,D) . For example, there are approximately 10 ATAC-seq peaks in the genome that are control-specific in 4 of the 6 matched pairs (see panel A).

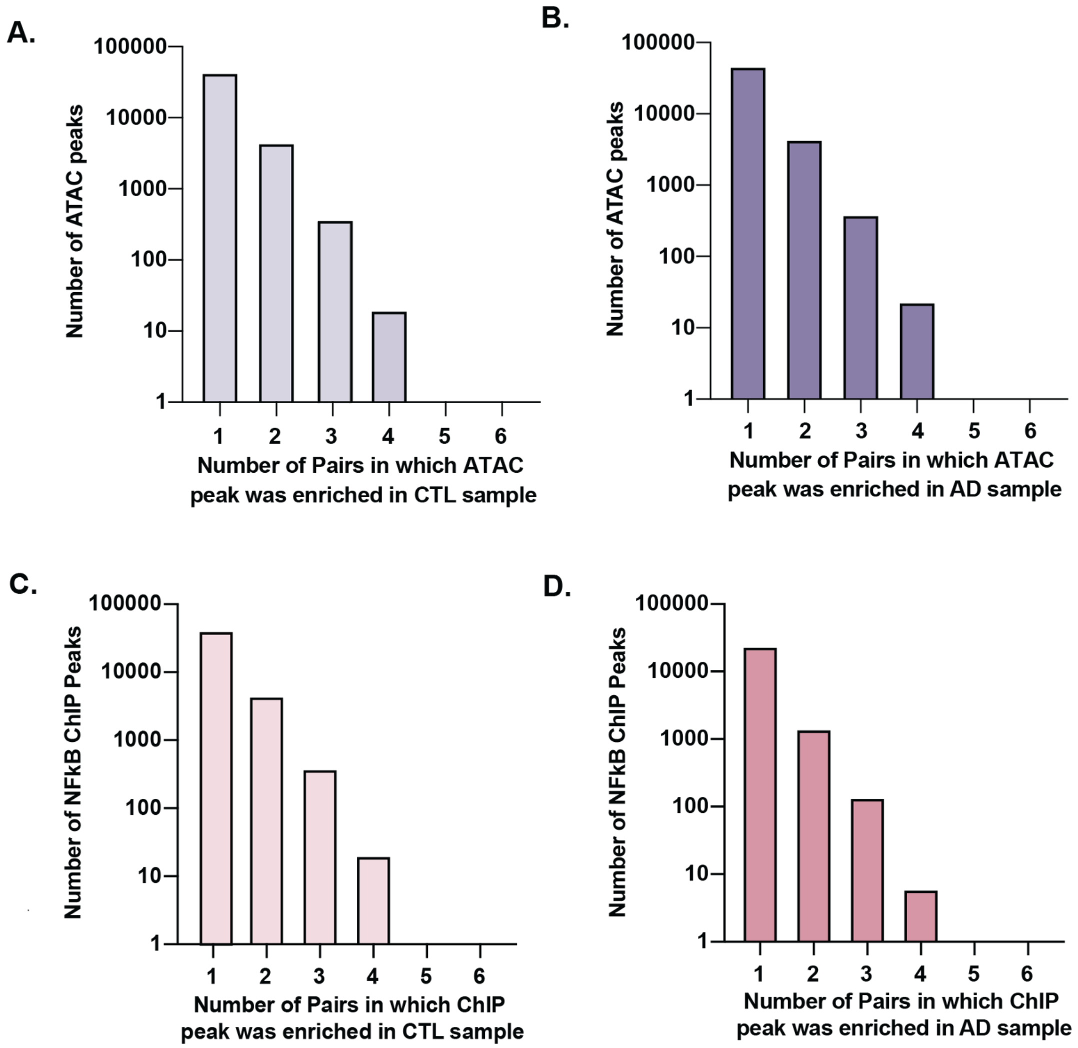

Supplement: S3 Fig — ATAC-seq and ChIP-seq peaks were called for all cases and controls. For each matched pair of samples, a MAnorm analysis identified shared peaks, control-specific peaks and AD-specific peaks (see Methods). Bar plots indicate the distributions of the number of control- specific (left) or case-specific peaks (right) for ATAC-seq (A,B) and ChIP-seq (C,D). For example, there are approximately 10 ATAC-seq peaks in the genome that are control-specific in 4 of the 6 matched pairs (see panel A). (PDF) [file pgen.1009973.s003.pdf]
