# Supplementary material for: Epigenetic and transcriptional dysregulation in CD4+ T cells in patients with atopic dermatitis
Source: PLoS Genet. 2022 May 16;18(5):e1009973. doi: 10.1371/journal.pgen.1009973 (PMC9135339; doi:10.1371/journal.pgen.1009973)

Supplement Figure 4. Case/control pairwise differential NKFB1 ChIP-seq analysis and transcription factor motif enrichment. (**Left plots**). For each of the six matched sample pairs, differential NKFB1 ChIP-seq peak analysis was performed. ChIP-seq peaks that are equally strong between case and controls (“shared”), significantly stronger in the control (“control-specific”), or significantly stronger in the case (“AD-specific”) were identified using MAnorm (see Methods). The percent of peaks falling within each category is indicated to the left of each heatmap. Heatmap color indicates the normalized strength of the ChIP-seq signal. (**Right plots**). Transcription factor motif enrichment analysis was performed for each sample pair (see Methods). Each dot represents a transcription factor motif. The X-axis and Y-axis indicate the p-value of enrichment within control-specific and AD-specific ChIP-seq peaks, respectively. Motif family color key is provided at the top of each plot.

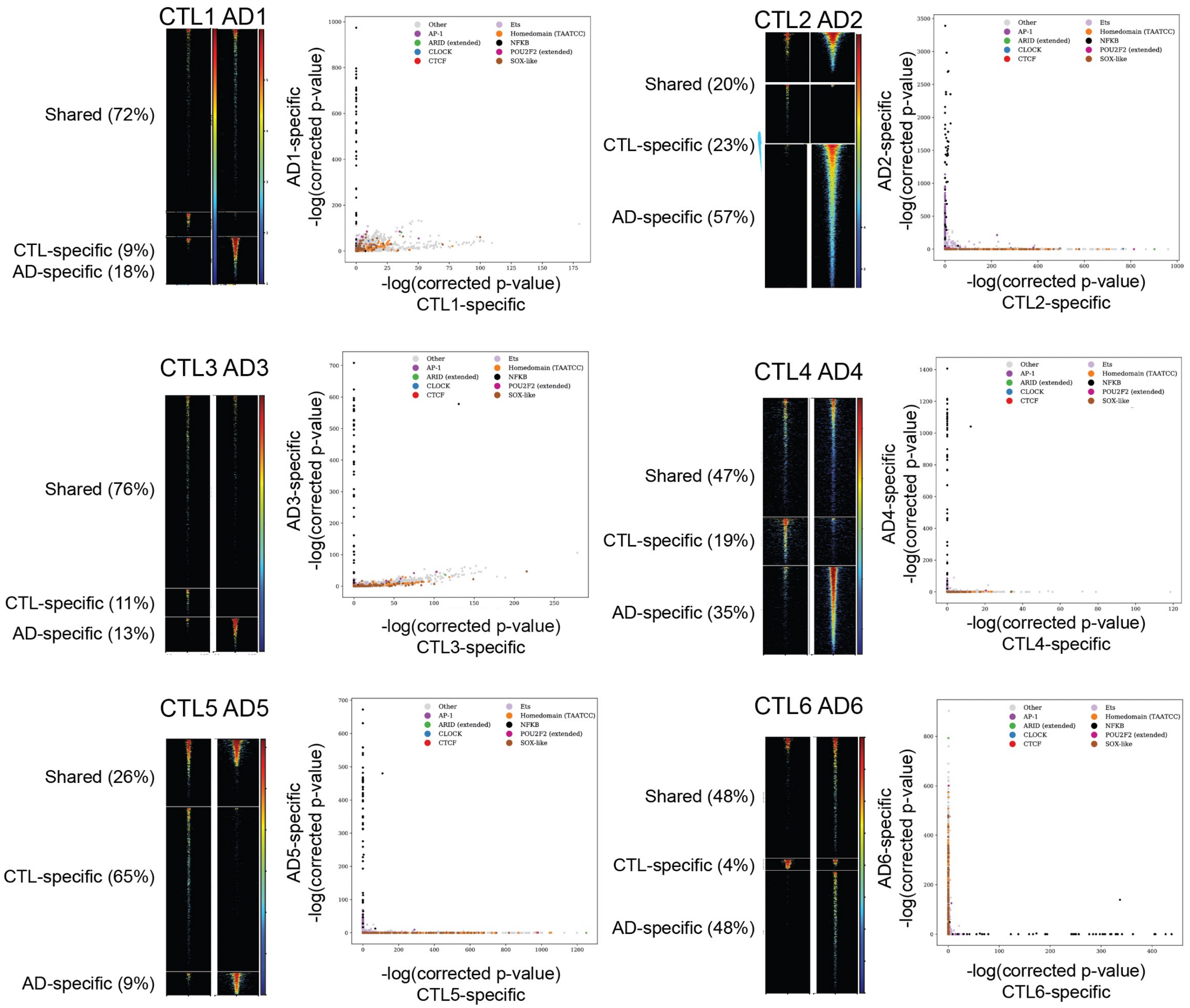

Supplement: S4 Fig — (Left plots). For each of the six matched sample pairs, differential NKFB1 ChIP-seq peak analysis was performed. ChIP-seq peaks that are equally strong between case and controls (“shared”), significantly stronger in the control (“control-specific”), or significantly stronger in the case (“AD-specific”) were identified using MAnorm (see Methods). The percent of peaks falling within each category is indicated to the left of each heatmap. Heatmap color indicates the normalized strength of the ChIP-seq signal. (Right plots). Transcription factor motif enrichment analysis was performed for each sample pair (see Methods). Each dot represents a transcription factor motif. The X-axis and Y-axis indicate the p-value of enrichment within control- specific and AD-specific ChIP-seq peaks, respectively. Motif family color key is provided at the top of each plot. (PDF) [file pgen.1009973.s004.pdf]
