# Supplementary material for: Epigenetic and transcriptional dysregulation in CD4+ T cells in patients with atopic dermatitis
Source: PLoS Genet. 2022 May 16;18(5):e1009973. doi: 10.1371/journal.pgen.1009973 (PMC9135339; doi:10.1371/journal.pgen.1009973)

Supplemental Figure 5: Transcription factor motif enrichment comparison between consistently control-specific and consistently AD-specific NFKB1 ChIP-seq peaks. The top five enriched TF motif families are shown. **A.** Percent of peaks containing predicted binding sites for the indicated motif. **B.** Motif enrichment p-value within those peaks (see Methods).

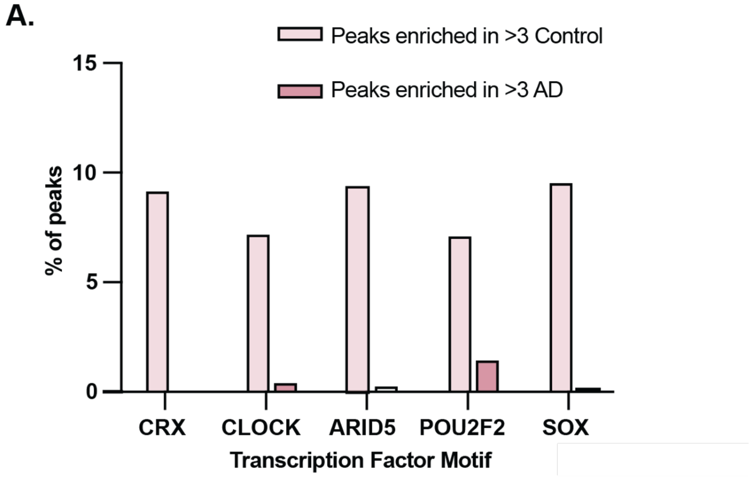

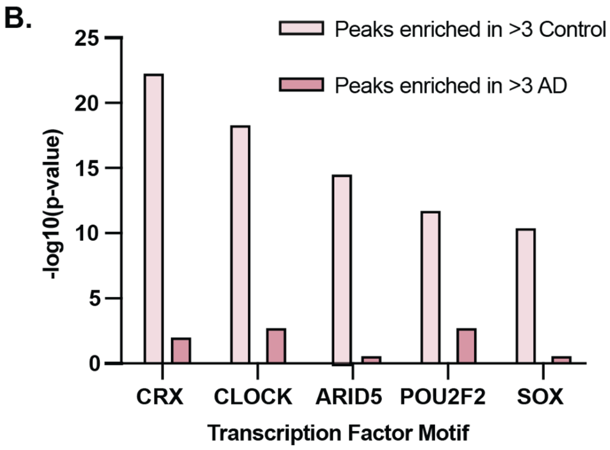

Supplement: S5 Fig — The top five enriched TF motif families are shown. A. Percent of peaks containing predicted binding sites for the indicated motif. B. Motif enrichment p-value within those peaks (see Methods). (PDF) [file pgen.1009973.s005.pdf]
