# Supplementary material for: Epigenetic and transcriptional dysregulation in CD4+ T cells in patients with atopic dermatitis
Source: PLoS Genet. 2022 May 16;18(5):e1009973. doi: 10.1371/journal.pgen.1009973 (PMC9135339; doi:10.1371/journal.pgen.1009973)
Supplement: S6 Fig — Pairwise differential gene expression analysis was performed by comparing RNA-seq data for each of the cases with their matched controls (see Methods). Genes with 1.5-fold changes or greater in three or more pairs were identified. Pathway enrichment analysis was performed on these gene sets. The top five enriched GO biological pathways and GO molecular function categories are indicated within the top and bottom tables, respectively. (PDF) [file pgen.1009973.s006.pdf]

Supplemental Figure 6. RNA-seq GO enrichment results. Pairwise differential gene expression analysis was performed by comparing RNA-seq data for each of the cases with their matched controls (see Methods). Genes with 1.5-fold changes or greater in three or more pairs were identified. Pathway enrichment analysis was performed on these gene sets. The top five enriched GO biological pathways and GO molecular function categories are indicated within the top and bottom tables, respectively.

Go: Biological Pathways

ID	Name	pValue	FDR B&H	Genes from Input	Genes in Annotation
GO:0002682	regulation of immune system process	1.01E-10	2.20E-07	16	1747
GO:0046649	lymphocyte activation	9.11E-09	9.91E-06	11	865
GO:0045321	leukocyte activation	1.66E-08	1.21E-05	13	1447
GO:0001775	cell activation	6.54E-08	3.44E-05	13	1623
GO:0019221	cytokine-mediated signaling pathway	7.89E-08	3.44E-05	10	82

Go: Molecular Function

ID	Name	pValue	FDR B&H	Genes from Input	Genes in Annotation
GO:0005126	cytokine receptor binding	3.24E-05	4.88E-03	5	295
GO:0031730	CCR5 chemokine receptor binding	4.44E-05	4.88E-03	2	8
GO:0050998	nitric-oxide synthase binding	5.90E-04	2.60E-02	2	28
GO:0061629	RNA polymerase II-specific DNA-binding transcription factor binding	6.34E-04	2.60E-02	4	312
GO:0005164	tumor necrosis factor receptor binding	8.71E-04	2.60E-02	2	34
