# Supplementary material for: Epigenetic and transcriptional dysregulation in CD4+ T cells in patients with atopic dermatitis
Source: PLoS Genet. 2022 May 16;18(5):e1009973. doi: 10.1371/journal.pgen.1009973 (PMC9135339; doi:10.1371/journal.pgen.1009973)
Supplement: S13 Table — (DOCX) [file pgen.1009973.s019.docx]

Supplemental Table 13. Demographics of participants in this study.

| **Patient ID** | **Age (years)** | **Patient Sex** | **Self-Reported Race** | **Self-Reported Ethnicity** |
| --- | --- | --- | --- | --- |
| AD1 | 8 | M | White | Not Hispanic |
| CTL1 | 10 | M | White | Not Hispanic |
| AD2 | 25 | F | White | Not Hispanic |
| CTL2 | 18 | F | White | Not Hispanic |
| AD3 | 12 | F | Black | Not Hispanic |
| CTL3 | 17 | F | Black | Not Hispanic |
| AD4 | 55 | M | White | Not Hispanic |
| CTL4 | 39 | F | White | Not Hispanic |
| AD5 | 67 | F | White | Not Hispanic |
| CTL5 | 53 | F | White | Not Hispanic |
| AD6 | 30 | F | White | Not Hispanic |
| CTL6 | 31 | F | White | Not Hispanic |
