# Supplementary material for: Epigenetic and transcriptional dysregulation in CD4+ T cells in patients with atopic dermatitis
Source: PLoS Genet. 2022 May 16;18(5):e1009973. doi: 10.1371/journal.pgen.1009973 (PMC9135339; doi:10.1371/journal.pgen.1009973)
Supplement: Attachment — Submitted filename: AD_response_to_reviewer_comments_v8_FINAL_2.pdf [file pgen.1009973.s020.pdf]

Reviewer #1: Thanks to the authors for this paper. I have only two criticisms:

1. The paper largely focuses on overlaps in genomic regions without applying any statistical evidence that those overlaps are more extreme than would be expected by chance alone. In the resubmission I would advise including hypergeometric testing or something similar to assess the strength of evidence.

We apologize for the confusion in the presentation of our work. Throughout the manuscript, we employ our computational tool Regulatory Element Locus Intersection (RELI) (Nat Genet. 2018;50(5):699-707. PMID: 29662164) to estimate the significance of the overlap between the genomic features employed in this study (TF binding events, histone marks, ATAC-seq peaks, etc.). As input, RELI takes the genomic coordinates of peaks from two datasets. RELI then systematically intersects these coordinates with one another, and the number of input regions overlapping the peaks is counted. Next, a p-value describing the significance of this overlap is estimated using a simulation-based procedure in which the peaks from the first dataset are randomly distributed within open chromatin regions from human cells (the "negative set"). A distribution of expected overlap values is then created from 2,000 iterations of randomly sampling from this negative set, each time choosing a set of negative examples that match the input set in terms of the total number of genomic loci. The distribution of the expected overlap values from the randomized data is modeled using a normal distribution and can thus be used to generate a Z-score and corresponding p-value estimating the significance of the observed number of input regions that overlap each dataset.

Since its initial introduction, RELI has been used to identify statistical enrichment for functional genomic datasets in numerous studies, including:

- Cell Metabolism. 2021;33(6):1187-204 e9. PMID: 34004162
- Allergy. 2021;76(6):1836-45. PMID: 33175399
- Nature Communications. 2021;12(1):1611. PMID: 33712590
- Nature Struct Mol Biol. 2020;27(10):978-88. PMID: 32895557.
- Nature Communications. 2021;12(1):567. PMID: 33495464.
- J Exp Med. 2020;217(1). PubMed PMID: 31653690.
- Genome Res. 2021. PMID: 34799401.
- PLoS Genet. 2021;17(6):e1009574. PMID: 34111109
- Blood. 2018;132(21):e24-e34. PMID: 30249787.
- eLife. 2020;9. PMID: 33006313.
- Cell Reports. 2021;34(12):108891. PMID: 33761354.

From our standpoint, this isexactly the kind of analysis that the reviewer is requesting, but actually slightly better, since RELI can control for things a simple hypergeomtric test cannot (e.g., the varying width of ChIP-seq peaks, and the non-uniform distribution of ChIP-seq peaks across the genome). We apologize for not making this clearer in the manuscript. To ensure that readers understand the analytical pipeline used to assess significance of overlapped datasets, we have added the following to the methods section on lines 546-560:

"Enrichment analysis for functional genomic datasets: We used the RELI algorithm to estimate the significance of the overlap between genomic features generated in this study (TF binding events, histone marks, ATAC-seq peaks, etc.). As input, RELI takes the genomic coordinates of peaks from two datasets. RELI then systematically intersects these coordinates with one another and the number of input regions overlapping the peaks is counted. Next, a p-value describing the significance of this overlap is estimated using a simulation-based procedure in which the peaks from the first dataset are randomly distributed within the union coordinates of open chromatin from human cells. A distribution of

expected overlap values is then created from 2,000 iterations of randomly sampling from the negative set, each time choosing a set of negative examples that match the input set in terms of the total number of genomic loci. The distribution of the expected overlap values from the randomized data resembles a normal distribution and can thus be used to generate a Z-score and corresponding p-value estimating the significance of the observed number of input regions that overlap each dataset."

2. The statement below is copied from the manuscript:

The 100 kB region of DNA around AD-specific gene sets widely overlapped (94.7-100%) the ATAC-seq peaks in the six subjects with AD (Supplemental Table 8). There was substantial overlap (26.3-68.4%) between the 100 kb region of DNA around ADspecific gene sets with the AD-specific ATAC-peaks, indicating that possible enhancers proximal to the AD-specific genes were accessible for transcription in an AD-specific manner.

The above gives no indication of the directionality of effect. Can you produce a graph or something similar to show the relationship between chromatin accessibilty and gene expression i.e logFC case v control (GE) v logFC case v control (ATAC)

We compared AD- and control-specific ATAC-seq peaks to AD- and control-specific genes (RNA-seq). Comparisons were done on a "matched subject pair" basis, using 100kb windows around the genes (e.g., ATAC-seq peaks found only in subject AD1 vs. CTRL1 were compared to genes with higher expression in AD1 vs CTRL1). We did observe a trend for AD-specific gene loci to more frequently contain AD-specific ATAC-seq peaks, although the difference are not significant (new Figure 5 below). We note that opening DNA that encodes a silencer can reduce gene expression, while closing a silencer can increase gene expression; thus, we do not expect perfect correlation between AD-specific ATAC-seq and AD-specific gene expression directionality.

There is a more striking finding from the ChIP-seq data. For all AD-specific gene sets (up in AD and down in AD), there is more overlap with NFKB1 in AD compared to control.

Figure 5

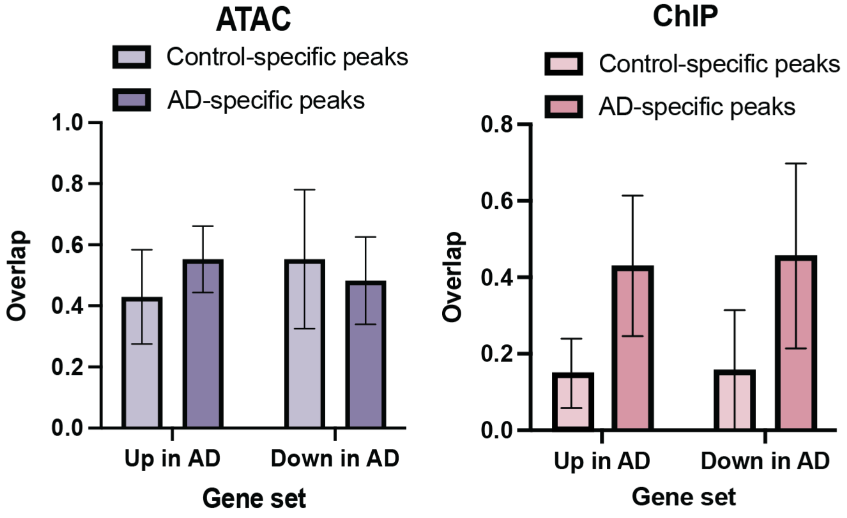

We have modified the manuscript accordingly (lines 306-324).

This section now reads: The 100 kB region of DNA around AD-specific gene sets widely overlapped (94.7-100%) the ATAC-seq peaks in the six subjects with AD (**Supplemental Table 8**). There was substantial overlap (26.3-68.4%) between the 100 kb region of DNA around AD-specific gene sets with

the AD-specific ATAC-peaks (**Figure 5**), indicating that possible enhancers proximal to the AD-specific genes were accessible for transcription in an AD-specific manner. Similarly, the 100 kb region of DNA around AD-specific NFKB1 ChIP-seq peaks overlapped the transcriptional start site of 47-95% of the AD-specific genes (**Supplemental Table 8, Figure 5**). In five of the six pairs, AD-specific NFKB1 ChIP-seq peaks overlapped a large proportion of the AD-specific genes (42.1-73.4%) (**Supplemental Table 8**). The 100 kb region around AD-specific NFKB1 ChIP-seq peaks overlapped 44.3% of the genes with increased expression in AD. In contrast, only 15.4% of genes with increased expression in AD had control-specific NFKB1 ChIP-seq peaks within 100 kb (**Figure 5**). Importantly, there is AD specificity to the overlap of NFKB1 ChIP-seq peaks, with AD-specific genes with AD-specific NFKB1 peaks overlapping more AD-specific genes (~80%) while control specific NFKB1 peaks overlapped only ~35% of AD-specific genes (**Figure 5**). These results are consistent with NFKB1 acting as both an activator and a repressor depending on the context (25)(26). Collectively, these data indicate strong agreement between AD- and control-specific gene expression, chromatin accessibility, and NFKB1 binding.

3. Likewise is it possible to include a figure demonstrating the allele-specific chromatin accessibility i.e ATAC reads stratified by genotype in cases and controls or something similar?

We have added red tick marks as an indication to Figure 5 to identify if the allelic ATAC was found in a case with AD or in a control. We do not find an increase in allelic behavior in cases relative to controls, which is consistent with previous studies of autoimmune diseases in which allelic behavior follows genotype and not case/control status:

- Nat Genet. 2018; 50(5):699-707. PMID: 29662164
- Nat Genet. 2018; 50(3): 424–431. PMID: 29379200
- Nature Communications. 2018; 9:2905. PMID: 30046115
- Science 2020; 369(6503):561-565. PMID: 32732423
- Genome Res. 2021. PMID: 34799401.

The finding is also consident with the presence of genotype-dependent expression (eQTLs) in non-disease cohorts (e.g. DICE or GTeX).

Please see the edited Figure below.

Figure 6

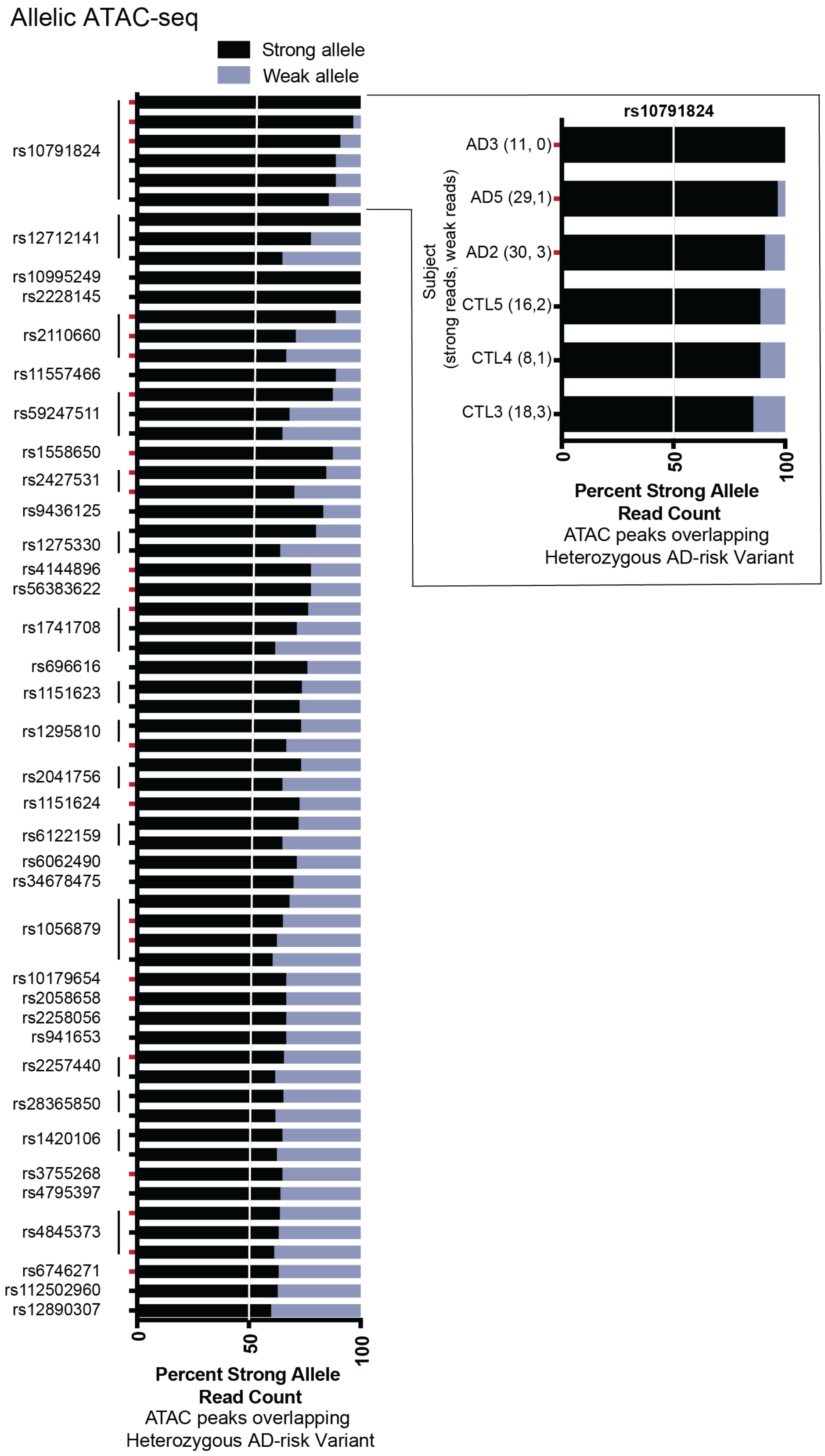

Allelic ATAC-seq
Strong allele
Weak allele
rs10791824
rs12712141
rs10995249
rs2228145
rs2110660
rs11557466
rs59247511
rs1558650
rs2427531
rs9436125
rs1275330
rs4144896
rs56383622
rs1741708
rs696616
rs1151623
rs1295810
rs2041756
rs1151624
rs6122159
rs6062490
rs34678475
rs1056879
rs10179654
rs2058658
rs2258056
rs941653
rs2257440
rs28365850
rs1420106
rs3755268
rs4795397
rs4845373
rs6746271
rs112502960
rs12890307
0
50
100
Percent Strong Allele Read Count
ATAC peaks overlapping Heterozygous AD-risk Variant
rs10791824
Subject (strong reads, weak reads)
AD3 (11, 0)
AD5 (29,1)
AD2 (30, 3)
CTL5 (16,2)
CTL4 (8,1)
CTL3 (18,3)
0
50
100
Percent Strong Allele Read Count
ATAC peaks overlapping Heterozygous AD-risk Variant

Reviewer #2: This is an interesting report from Eapen et al., that characterizes the epigenetic and transcriptional dysregulation of CD4 T cell in patients with atopic dermatitis (AD). AD affects approximately 20% of children and high rate of persistence into adulthood. There are 29 independent risk haplotypes identified. It is well know that CD4 T cells are the major effector cell type for AD and that NFKB signaling mediates the pathogenic inflammation.

This paper seeks to determine if there are upstream effects in CD4 T cells at the level of epigenome and transcriptome changes that facilitate pathogenic T cell responses. The authors use a combination of genomic techniques to determine if AD risk haplotypes demonstrate altered chromatin accessibility, NFKB binding and gene expression changes consistent with AD using a case/control study design in CD4 T cells in patients with AD. They demonstrate that in stimulated CD4 T cells taken from patients with AD that open chromatin regions were enriched for AD risk variants and that there was strong enrichment for NFKB binding motifs in these peaks in AD patients but not in controls.

They also demonstrated over 60 instances of genotype-dependent chromatin accessibility for 36 AD risk variants. Together they conclude that allele specific epigenetic and transcriptional regulation is an important feature of CD4 T cell responses to stimulation in patients with AD. While these results are interesting, there are several significant issues that need to be addressed to improve the overall quality of this manuscript. These are listed below in no specific order of importance:

1. The text is missing some basic proofreading

We submitted this manuscript to the copy editor service "Editage" for advanced editing and incorporated over 95% of their suggested changes. Invoice is provided below.

editage

CONTACT US
Email: payments@editage.com
Phone: +1 (833) 979-0061

RECEIPT

To
Dr. Leah Kottyan
United States
Phone: +1 5136361316
Email: leah.kottyan@cchmc.org

From
Cactus Communications Services Pte. Ltd.
20 McCallum Street,
#19-01,
Tokio Marine Centre Singapore - 069046
Phone: +1 (833) 979-0061
GST Reg. No.: 201526201R

Receipt detail	
Client code	**LETTY**
Client name	Dr. Leah Kottyan
Organisation name	
Payment mode	Stripe - ENG
Payment date	6 Mar 2022
**Total received**	**$448.47**

Cactus Communications Services Pte. Ltd.
20 McCallum Street, #19-01, Tokio Marine Centre Singapore - 069046
Phone: +1 (833) 979-0061, www.cactusglobal.com
United States • Japan • India • South Korea • China • Singapore

In total, we accepted ~500 changes from the copy editor.

2. Testing the hypothesis that "AD loci may be epigenetically regulated" is vague (pp. 2, 128).

The text has been edited as follows (line 132-133):

The original sentence in the Introduction: "Herein we hypothesized that AD loci may be epigenetically regulated".

Revised sentence: "In this study, we hypothesized that the chromatin accessibility and binding of transcription factors may regulate genetic AD risk loci."

3. The FRiP scores are quite low with average of 32% indicating that the ATAC-seq data may have QC issues (typically >50% is expected), please address.

The current ENCODE standards for ATAC-seq data (https://www.encodeproject.org/atac-seq/#standards) state that "The fraction of reads in called peak regions (FRiP score) should be >0.3, though values greater than 0.2 are acceptable. For EN-TEx tissues, FRiP scores will not be enforced as QC metric. TSS enrichment remains in place as a key signal to noise measure."

Thus, the official ENCODE policy is to have FRiPs ideally exceeding 0.3 for experiments performed in cell lines, with 0.2 still being acceptable. Note that for studies such as ours, which were performed in primary cells, they do not require a minimum FRiP score. Nevertheless, all but one of our ATAC-seq experiments have FRiP scores > 0.2, with a mean of 0.3.

4. Analysis of shared peaks was performed by using pairwise assessments. It is not clear how case/control pairs were selected and why a more exhaustive case/control analysis was not performed (i.e., pairwise comparisons using all possible pairs)

We matched each pair of samples by age and ancestry. At the beginning of the study we identified heterogeneity across samples, consistent with literature demonstrating differences within the CD4+ immune compartment as a person ages (eg Immun Aging. 2019 Sep 11;16:24. PMID: 31528179 and Clinical Experimental Immunology 2021 Dec 29; PMID: 3502089). We thus did not compare across all samples because of the heterogeneity that was introduced by different sex, ancestries, and ages. Instead, we choose to focus on the matched case:control pairs to facilitate the identification of consistent differences between AD and controls.

We have now included **Supplemental Table 13**, which describes the patient – control matching characteristics in detail.

Patient ID	Age (years)	Patient Sex	Self-Reported Race	Self-Reported Ethnicity
AD1	8	M	White	Not Hispanic
CTL1	10	M	White	Not Hispanic
AD2	25	F	White	Not Hispanic
CTL2	18	F	White	Not Hispanic
AD3	12	F	Black	Not Hispanic
CTL3	17	F	Black	Not Hispanic
AD4	55	M	White	Not Hispanic
CTL4	39	F	White	Not Hispanic
AD5	67	F	White	Not Hispanic
CTL5	53	F	White	Not Hispanic
AD6	30	F	White	Not Hispanic
CTL6	31	F	White	Not Hispanic

We have also added a section to the Discussion focused on the importance of further controlling for age and ancestry in future studies to allow for pooled analysis (line 388):

"This study used a paired analysis of cases with demographically matched controls. The findings of this manuscript represent epigenetic differences that are consistent across these matched pairs. Future studies will be needed in which ancestry and age are carefully matched for all cases and controls to support a pooled analytical strategy."

5. More generally, how were subjects matched?

See above. Age and self-reported race and ethnicity.

6. After the pairing approach used to identify AD- or control-specific ATAC peaks, peaks that were AD- or control-specific in 3 or more of the pairings were classified as "consistently AD/control" (pp. 5, 176-178). It is not stated how much these peak sets overlap each other. Based on the data in the supplement (Supplemental Table 3), they share 80% of LD SNPs so it is likely they have some overlap.

We define "consistent" as being present in three or more cases (AD consistent) or controls (CTL consistent). This is why there is large overlap between AD consistent and CTL consistent peaks – the vast majority of open chromatin regions in the genome do not change from individual to individual as a function of disease status.

We define "specific" based on MANORM statistical differential analysis. Consistently AD-specific peaks are statistically stronger in AD in three or more pairs. Consistently control-specific peaks are statisticaly stronger in control subjects in three or more pairs. Consistently AD-specific ATAC peaks have no overlap with consistently CTL-specific ATAC peaks.

To clarify this finding in the Results section, we have added the following text to line 188-194:

"As expected, there was substantial overlap between AD-consistent and control-specific ATAC-seq peaks since most chromatin states do not change from individual to individual within a cell type. We identified 5,639 AD-specific consistent ATAC-seq peaks (i.e., consistent peaks that were not consistent in controls). Likewise, we identified 10,065 control-specific consistent ATAC-seq peaks. Consistently AD-specific ATAC peaks have no overlap consistently control-specific ATAC peaks."

Please see the answer below for the question around the overlapped AD risk variants.

7. Based on shared LD and Tag SNPs (Supplemental Table 3) it is NOT clear that chromatin is accessible in a "disease specific manner" (pp. 5, 181-182). Twelve of the 13 AD risk loci overlapped by "consistently AD" peaks are also overlapped by "consistently control" peaks.

Thank you for pointing this out. In the context of the overlap between AD-specific ATAC and CTL-specific ATAC, we have added a sheet to STable 3 to highlight the number of AD-risk variants overlapping AD-specific and CTL-specific ATAC peaks.

In this sheet (also presented below), the overlapping SNP is presented in the first column. In the second column, the Tag SNP is provided so that readers can identify instances in which multiple SNPs in the same haplotype overlap with ATAC-seq peaks. There are 24 AD risk variants in 17 haplotypes that

overlap consistent AD ATAC-peaks. Of these, five overlap consistent AD ATAC-seq peaks but not consistent CTL peaks.

SNP inside ATAC-seq peak (LD SNP)	locus ID (Tag SNP)	Consistently AD (present in 3 or more samples)	Consistently CTL (present in 3 or more samples)
rs11602467	rs12295535	YES	NO
rs6684439	rs2228145	YES	NO
rs2058658	rs6419573	YES	NO
rs1291209	**rs909341**	YES	NO
rs1295810	**rs909341**	YES	NO
rs6890853	rs10214237	YES	YES
rs10791824	rs10791824	YES	YES
rs9391734	rs12153855	YES	YES
rs77190366	rs12295535	YES	YES
rs112502960	rs16948048	YES	YES
rs17389644	rs17389644	YES	YES
rs1048990	**rs2143950**	YES	YES
rs28365850	**rs2143950**	YES	YES
rs4845373	rs2228145	YES	YES
rs1420106	rs6419573	YES	YES
rs10957979	**rs6473227**	YES	YES
rs11776367	**rs6473227**	YES	YES
rs2370615	**rs6473227**	YES	YES
rs4739737	**rs6473227**	YES	YES
rs5892724	**rs6473227**	YES	YES
rs11236797	**rs7110818**	YES	YES
rs34455012	**rs7110818**	YES	YES
rs4144896	rs7127307	YES	YES
rs2427531	rs909341	YES	YES

In addition to the text added in response to Reviewer 2 Question 6, we have also added the following sentence to the results:

"In total, we identified 361 consistently AD-specific ATAC-seq peaks."

"24 AD risk variants in 17 haplotypes overlap ATAC-peaks consistently found in subjects with AD. Of these, five overlap ATAC-seq peaks consistently found in subjects with AD but not consistently found in controls."

We have also added the table above in a sheet in Supplemental Table 3.

8. On pp. 5, 170-171, authors state 75-88.4% of ATAC peaks were shared between AD and demographically matched controls, but on pp. 8, 231 authors state a median of 91.9% of ATAC peaks were shared. It is unclear how this inconsistency arose or if the comparison being made is different in the second statement in a way that is not clear from the text.

The numbers reported in the manuscript were not correct. The data in Figure 3 were correct. The sentence has been corrected as follows (now line 178):

Original: In pairwise assessments performed using MAnorm (24), most ATAC-seq peaks were shared between AD patients and demographically matched controls (75-88.4%).

Corrected: In pairwise assessments performed using MAnorm (24), most ATAC-seq peaks were shared between AD patients and demographically matched controls (85.9-96.0%).

The median of 91.9% was accurate.

9. On pp. 22, 471-472 in the METHODS section, authors state that reads for ChIP-seq were processed the same way as ATAC-seq reads, but the MACS2 parameters needed to properly call peaks in these two data types should be different to avoid errors in the genomic coordinates of the called peaks, especially excluding parameters '-nomodel --shift --extsize' for ATAC-seq peak calling.

Many different approaches are used for peak calling. Because there is currently no single established gold standard method, we try to follow ENCODE recomendations whenever possible.

For ATAC-seq and ChIP-seq, ENCODE's suggested parameters are as follows:

ATAC-seq: macs2 callpeak --nomodel --shift -75 --extsize 150
ChIP-seq: macs2 callpeak --nomodel --shift 0 --extsize fraglen

The difference between the two methods is the "--shift -75 --extsize 150" recommended for ATAC-seq. These parameter settings shift the reads 75 base pairs from the 3' → 5' direction, and then extend the size of the read in the 5' → 3' direction to create a fragment of length 150bp centered around the place where the read originally mapped.

For our analysis, we used the following in macs2:
macs2 callpeak --q 0.01

In other words, we used the most commonly used peak calling algorithm (macs2) with default settings. We also used a 50bp padding to extend the called peaks on both sides which help amplify the cutting site enrichment.

Until Fall of 2021, our laboratory used a pipeline that we called "NextGenAligner" to call peaks and run quality assessment on next generation datasets. We are in the process of shifting to the ENCODE pipeline, which is non-trivial (one year in, with the involvement of three postdocs, we are almost there). Based on this Reviewer comment, we reran all of the ATAC-seq data through our preliminary ATAC-seq ENCODE pipeline. To confirm that the differences in peak calling do not affect the findings of this manuscript, we re-ran HOMER motif enrichment analysis and found exceptional concordance between motif enrichment for the two peak calling methods:

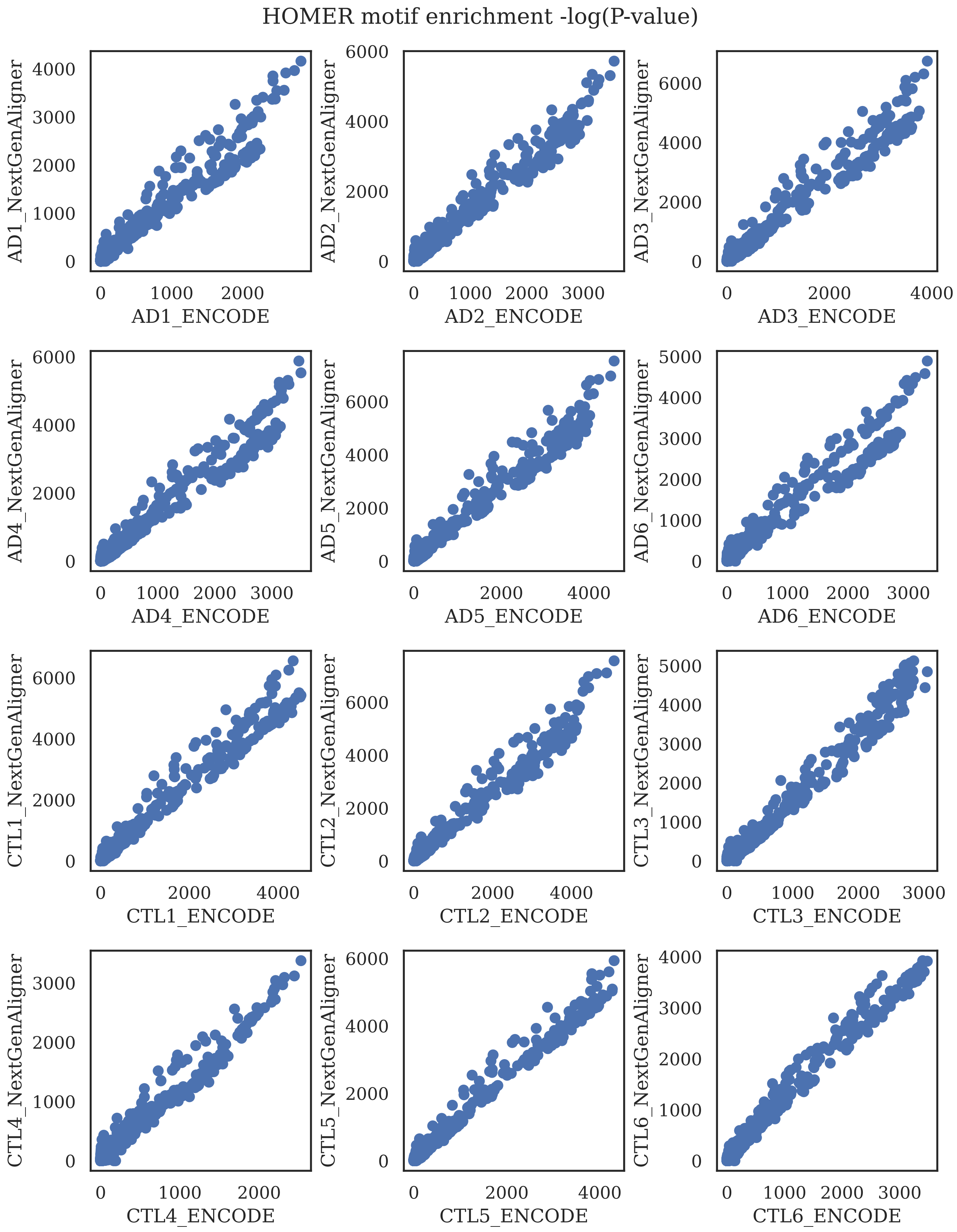

Figure R1. HOMER TF motif enrichment comparison between peak calling methods employed in this study (Y-axis) and by the ENCODE pipeline (X-axis). Each point represents a human TF binding site motif (obtained from the Cis-BP database). The X- and Y-axis indicate the -log p-value of the enrichment of that motif in the corresponding peak set.

Please also see the "TSS enrichment" results presented in Supplemental Table 2 – the ATAC-seq peaks called using our method clearly meet ENCODE standards for each subject, with a mean TSS enrichment of 19.5 (compared to 21.5 using our preliminary implementation of the ENCODE pipeline). (ENCODE considers TSS enrichment scores over 10 as "ideal"). We thus conclude that our peak calling did not affect the conclusions in this manuscript.

10. On pp. 8, 228-231, it states that there is more variability in overlap between subject matched pairs for ChIP vs. ATAC peaks, but this may be entirely due to the fact that there is substantial variability in the number of ChIP peaks across samples (Supplemental Table 2). It would be useful to compare variability of overlap within controls and within AD to see if the variability between control vs. AD is any greater than the large variability already present in the samples.

Please see the variability analysis of ChIP and ATAC broken down by case:control status.

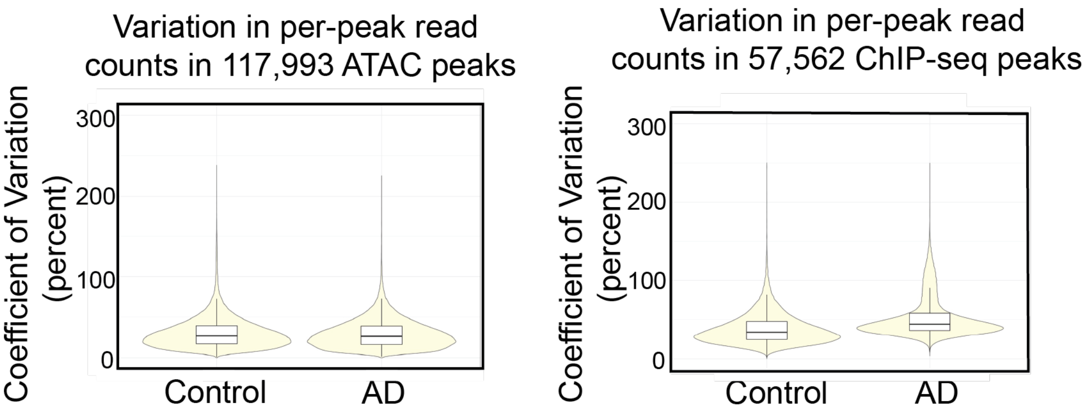

This analysis has been added as a subfigure in to Supplemental Figure 1. The following statement has been added to the results:

"A global variability analysis of ATAC and ChIP peaks revealed minimal differences in variability within cases versus within controls."

11. The biological processes associated with the differentially expressed genes (15 are expressed 1.5x higher in AD, 16 are expressed 1.5x lower in AD; shown in a table in Supplemental Figure 6) are not particularly compelling. These seem like very high-level and general processes and many of the enriched pathways include hundreds or even over 1000 genes. More generally, only finding 31 differentially expressed genes seems surprisingly low.

We agree that this study was not powered to robustly identify differential gene expression. When we take the list of genes that had a 1.5-fold difference between at least 2 of the 6 pairs (instead of 3), we identify 131 total genes, with a more robust set of 36 enriched pathways (adjusted p-values less than 0.0001 with at least four overlapping genes in the gene set). For example, the top pathway is IL2 signaling. IL2 is the cytokine that maintains T cell viability and survival during inflammatory responses. Indeed, each of the pathways with adjusted p-values less than 0.0001 are related to inflammatory signaling and T cells. The top five enriched pathways with at least 9 gene overlaps are shown below.

Term	Overlap	P-value	Adjusted P-value	Odds Ratio	Genes
Interleukin-2 signaling pathway	36/847	3.27E-21	1.47E-18	10.08	FURIN;SOCS3;MT2A;MYC;CCL4;UCP2;PIM1;PMAIP1;CCR7;TNFRSF4;S100A11;CTSC;IL12RB2;CD53;CD52;JUND;GPX4;STAT1;STAT3;ASNS;GZMB;EMP3;CD40LG;IFNG;IRF4;CD5;PSAT1;ID2;STK17B;BHLHE40;IL2RB;LTA;PTPN6;LY6E;BCL2L1;BIRC3
T cell receptor regulation of apoptosis	22/603	9.57E-12	2.15E-09	7.46	SERPINB1;JUND;STAT1;GZMB;ITGAL;DYNLL1;PRDX2;CCND3;LGALS1;CD40LG;IFNG;IRF4;SCD;MYC;STK17B;CCL4;PIM1;LTA;PMAIP1;CCR7;LAP3;BIRC3
Interferon-gamma signaling pathway	9/97	6.83E-09	7.66E-07	18.24	SOCS3;MT2A;IFNG;IRF4;OAS2;STAT1;STAT3;PTPN6;HLA-A
Jak-STAT signaling pathway	11/199	3.24E-08	2.91E-06	10.57	SOCS3;CCND3;IFNG;STAT1;MYC;STAT3;IL2RB;PIM1;PTPN6;BCL2L1;IL12RB2
Interferon signaling	10/168	7.01E-08	5.25E-06	11.35	EIF4A2;SOCS3;MT2A;IFITM2;IFNG;IRF4;OAS2;STAT1;PTPN6;HLA-A

We respectfully request not to share these results in the manuscript, as we do not think that fold-change differences in 2/6 comparisons is sufficient to report in a manuscript. It is also inconsistent with our choice to do 3/6 for the other data types. This manuscript provides the data needed to perform power calculations that will optimize future studies with sufficient statistical power to replicate and identify differences in gene expression from a similar study design. This opportunity in addition to the learned and shared experience in terms of the need to reduce noise through controlling for sex and ancestry will be important for future studies.

12. The case/control RNA-seq comparisons were only performed pairwise (pp. 11, 280), which seems like a missed opportunity for comparing pooled case vs. pooled control data, which would presumably have more power to detect differentially expressed variants (this applies to the pairwise comparisons used more generally)

As presented in response to the question above regarding pooling and case:control differences, we were frankly surprised by the age-dependent heterogeneity in the epigenetic measurements. Based on our findings, we do not want to introduce a demographic-dependent bias in the RNA analysis. In other words, if we are not able to pool the ATAC-seq or ChIP-seq data, we do not want to present pooled RNA-seq data. We have added a section to the Discussion (line 388) highlighting this limitation and encouraging future functional genomic studies of atopic dermatitis to control for demographics.

"This study used a paired analysis of cases with demographically matched controls. The findings of this manuscript represent epigenetic differences that are consistent across these matched pairs. Future studies will be needed in which age, sex, and ancestry are carefully matched for all cases and controls to support a pooled analytical strategy."

13. On pp. 12, 289-299, authors describe how a large % of AD-specific ATAC peaks overlap the 100kb region around AD-specific gene sets. They then state that NFKB1 ChIP-seq peaks overlap a large % of the TSS of AD-specific genes (they don't state whether it is AD-specific NFKB1 peaks or ALL peaks).

We only report AD-specific ATAC and AD-specific ChIP in Suplementary Table 8. We apologize for the confusion – we have modified the text to make this clearer.

They then state that a lower % of AD-specific NFKB1 peaks overlap the AD-specific genes (does this mean that the peaks overlap the transcripts?).

This means that the peaks are within 100 kb of the NFKB1 ChIP-seq peak. We have modified the text to make this clearer.

It would be helpful to show additional comparisons here to make the argument that control- and AD-ChIP/ATAC peaks are truly specific for control- and AD-genes. For example, what % of AD-specific genes show control-specific ATAC peaks in the 100kb vicinity? Is it similar to the % of AD-specific ATAC peaks, or is the % of AD-specific ATAC peaks significantly higher in the vicinity of AD-specific genes, etc.

Please see the analysis that was also provided for Reviewer 1 Question 2.

Figure 5

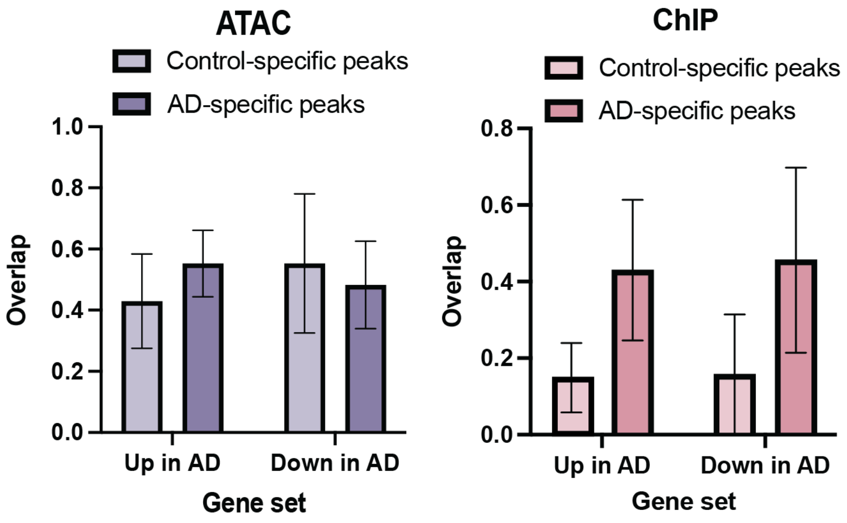

We interpret these results as indicating that AD-specific open chromatin tends to lead to higher expression in AD, and AD-specific closing of chromatin tends to lead to lower expression in AD. NFKB1 has a different overall trend, which likely indicates that AD-specific NFKB1 peaks can result in either AD-specific activation or repression of gene expression, depending on the genomic context of the NFKB1 binding event.

We have now added the overlap of AD and CTL-specific ATAC and NFKB datasets and up-regulated and down-regulated genes as a sheet in **Supplemental Table 8**. Additionally, we now show the overlap of 100 kb around AD and CTL-specific NFKB1 ChIP-seq with up and down regulated genes in **Figure 5**.

In text edits **(starting on line 306):**

Previously, this section stated: The 100 kB region of DNA around AD-specific gene sets widely overlapped (94.7-100%) the ATAC-seq peaks in the six subjects with AD (**Supplemental Table 8**). There was substantial overlap (26.3-68.4%) between the 100 kb region of DNA around AD-specific gene sets with the AD-specific ATAC-peaks, indicating that possible enhancers proximal to the AD-specific genes were accessible for transcription in an AD-specific manner. Similarly, the 100 kb region of DNA around NFKB1 ChIP-seq peaks overlapped the transcriptional start site of 47-95% of the AD-specific genes (**Supplemental Table 8**). In five of the six pairs, AD-specific NFKB1 ChIP-seq peaks overlapped a large proportion of the AD-specific genes (42.1-73.4%) (**Supplemental Table 8**).

Collectively, these data indicate strong agreement between AD- and control-specific gene expression, chromatin accessibility, and NFKB1 binding.

In response to this review, we have edited this section to read: The 100 kB region of DNA around AD-specific gene sets widely overlapped (94.7-100%) the ATAC-seq peaks in the six subjects with AD (**Supplemental Table 8**). There was substantial overlap (26.3-68.4%) between the 100 kb region of DNA around AD-specific gene sets with the AD-specific ATAC-peaks (**Figure 5**), indicating that possible enhancers proximal to the AD-specific genes were accessible for transcription in an AD-specific manner. Similarly, the 100 kb region of DNA around AD-specific NFKB1 ChIP-seq peaks overlapped the transcriptional start site of 47-95% of the AD-specific genes (**Supplemental Table 8, Figure 5**). In five of the six pairs, AD-specific NFKB1 ChIP-seq peaks overlapped a large proportion of the AD-specific genes (42.1-73.4%) (**Supplemental Table 8**). The 100 kb region around NFKB1 ChIP-seq peaks specific in AD overlapped 44.3% of the genes with increased expression in AD compared to the 15.4% of genes with increased expression in AD that were overlapped by 100 kb region around NFKB1 ChIP-seq peaks specific for controls (**Figure 5**). Importantly, there is AD specificity to the overlap of NFKB1 ChIP-seq peaks with AD-specific genes with AD-specific NFKB1 peaks overlapping more AD-specific genes (~80%) while control specific NFKB1 peaks overlapped only ~35% of AD-specific genes (**Figure 5**). These results are consistent with NFKB acting as both an activator and repressor depending on the context (25)(26). Collectively, these data indicate strong agreement between AD- and control-specific gene expression, chromatin accessibility, and NFKB1 binding.

14. Authors used MARIO method to integrate information across ATAC/ChIP/RNA-seq data with WGS for each of their subjects and find allele dependence of sequencing reads (read depth?) at het variants (het across the sample, or within individuals?) (pp. 13, 304-307)

As noted by the reviewer, functional genomic data (such as ATAC-seq and ChIP-seq) provide a means to identify allele-dependent chromatin accessibility and protein binding events on a genome-wide scale. In cases where a given variant is heterozygous in the cell assayed, both alleles are available for the TF to bind or for the chromatin to be accessible or not, offering a natural control for one another since the only variable that has changed is the allele. We previously developed the MARIO (Measurement of Allelic Ratios Informatics Operator) pipeline to identify allele-dependency by weighing imbalance between the number of sequencing reads for each allele of a given genetic variant, the total number of reads available at the variant, and the number and consistency of available experimental replicates (see Nat Genet. 2018;50(5):699-707. PMID: 29662164). MARIO is an easy-to-use, modular tool that (1) calculates a score that explicitly reflects reproducibility across experimental replicates; (2) reduces run-time via utilization of multiple computational cores; and (3) allows the user to directly provide genotyping data as input.

To estimate the significance of the degree of allelic imbalance of a given dataset at a given heterozygote, we developed the MARIO Allelic Reproducibility Score (ARS), which is based on a combination of two *predictive variables*: the total number of reads overlapping the variant and the imbalance between the number of reads for each allele. Other variables tested were uninformative (see Nat Genet. 2018;50(5):699-707. PMID: 29662164).

For the MARIO results reported in this paper, all results have MARIO ARS values > 0.4 and are hence allele-dependent according to previously published standards.

Currently, the following section is in the methods section where the MARIO analytical pipeline reads: <u>Identification of allelic ATAC-seq and ChIP-seq reads using MARIO</u>: To identify possible allele-dependent mechanisms in our functional genomics datasets, we applied our MARIO method [21]. In brief, MARIO identifies common genetic variants that are (1) heterozygous in the assayed cell line

(using NGS DNA sequencing data) and (2) located within a peak in a given ChIP-seq or ATAC-seq dataset. MARIO then examines the sequencing reads that map to each heterozygote within each peak for imbalance between the two alleles. We report allelic accessibility and NFKB1 binding at AD genetic risk variants in our ATAC-seq data with an Allelic Reproducibility Score (ARS) greater than or equal to 0.4 which is considered significantly allelic [21].

We have updated this section as follows to provide more information: Identification of allelic ATAC-seq and ChIP-seq reads using MARIO: To identify possible allele-dependent mechanisms in our functional genomics datasets, we applied our MARIO method [21]. In cases where a given variant is heterozygous in the cell assayed, both alleles are available for the TF to bind or for the chromatin to be accessible or not, offering a natural control for one another since the only variable that has changed is the allele. In brief, MARIO identifies common genetic variants that are (1) heterozygous in the assayed cell line (using NGS DNA sequencing data) and (2) located within a peak in a given ChIP-seq or ATAC-seq dataset. MARIO then examines the sequencing reads that map to each heterozygote within each peak for imbalance between the two alleles. To estimate the significance of the degree of allelic imbalance of a given dataset at a given heterozygote, we developed the MARIO Allelic Reproducibility Score (ARS), which is based on a combination of two *predictive variables*: the total number of reads overlapping the variant and the imbalance between the number of reads for each allele. We report allelic accessibility and NFKB1 binding at AD genetic risk variants in our ATAC-seq data with an ARS greater than or equal to 0.4 which is considered significantly allelic [21].

15. Pp. 13, 311-313: The authors state that the MARIO analysis discovers AD-associated variants in ATAC peaks that are het in some subjects and produce allele-dependent ATAC peaks. This seems like a circular argument: the MARIO analysis uses differences in sequencing reads combined with genotyping data to discover alleles that affect sequencing reads, so why is it striking that it finds alleles that produce allele dependent ATAC peaks, since that is the very signal it is based on?

Please see above. The reason that it is not circular is that the genome is masked for common variants before remapping all reads – thus the allelic differences in the reference genome do not affect read mapping/peak calling. It is important to note that the vast majority (~98%) of peaks overlapping a heterozygous variant did not have a significant allelic read imbalance.

16. Fewer AD-associated NFKB1 binding variants were found, but this may be largely driven by the fact that there were ~7x more ATAC peaks than ChIP peaks (as the authors acknowledge pp. 14, 328-329).

We agree. We have also added this limitation to the Discussion (lines 396).

"The paucity of AD-associated NFKB1 binding variants could in large part be due to the lower number of NFKB1 ChIP-seq peaks."

17. It would be interesting search the AD-associated alleles in large public datasets and look analyze their frequencies, whether they have been associated with other diseases, etc.

The allele frequencies and diseases associations are now presented in Supplemental Table 1:

Variation ID	Chromosome	Position	Reference Allele	Minor Allele	Risk Allele	Minor Allele Global Frequency	Odds Ratio
rs10199605	2	8495097	G	A	G	0.24	0.93
rs10214237	5	35883734	T	C	T	0.18	0.93
rs10738626	9	22373457	C	C	T	0.40	0.81
rs10791824	11	65559266	A	G	A	0.49	1.12
rs112111458	2	71100105	A	G	A	0.22	0.91
rs12153855	6	32074804	T	C	T	0.13	1.58
rs12295535	11	36432024	C	T	T	0.05	1.68
rs1295686	5	131995843	T	T	T	0.42	1.22
rs16948048	17	47440466	A	G	G	0.29	1.05
rs17389644	4	123497697	G	A	A	0.12	1.21
rs2041733	16	11229589	T	T	T	0.50	0.92
rs2143950	14	35572357	C	T	T	0.22	1.08
rs2228145	1	154426970	A	C	C	0.29	1.08
rs2918307	19	8789722	A	G	G	0.17	1.12
rs4713555	6	32575524	G	T	G	0.30	0.91
rs4809219	20	62303115	C	C	C	0.27	0.90
rs61813875	1	152536650	C	G	G	0.00	1.61
rs6419573	2	103027103	T	T	T	0.31	1.11
rs6473227	8	81285892	C	A	C	0.47	0.93
rs6602364	10	6038853	G	G	G	0.49	1.08
rs6720763	2	167992286	T	C	C	0.32	1.29
rs7110818	11	76292575	C	T	T	0.39	1.28
rs7127307	11	128187383	T	C	C	0.49	0.94
rs7130588	11	76270683	A	G	G	0.22	1.29
rs72702813	1	152600854	G	T	T	0.01	2.06
rs7512552	1	150265704	T	T	T	0.27	0.93
rs759382	2	103094213	G	G	G	0.31	1.22
rs848	5	131996500	A	A	A	0.37	1.40
rs909341	20	62328742	C	T	T	0.26	1.32

Regarding the disease associations beyond AD - we did not have room in this Reviewer Response to include them in the table presented above, but we did include them in the updated Supplemental Table 1. The additional associated phenotypes included expected phenotypes such as asthma.

The following sentence has been added to the Methods on line 440:

"Supplemental Table 1 presents the twenty-nine AD risk loci that reached genome-wide significance at the beginning of this study; this supplemental table includes information regarding the major, minor, reference, non-reference, and risk alleles as well as allele frequencies, disease odds ratios, and additional associated diseases and phenotypes."

To assess phenotypes related to the entire set of AD risk vairants, we also performed a PheWAS for the AD risk alleles using a database of patients with both genome-wide association data and electronic

medical records. This analysis of statistical significance (the red line marks the level of significance to overcome the multiple testing correction) identified shared genetic risk with asthma, eosinophilic esophagitis, and atopic dermatitis (AD). The association with AD is a positive control for the analysis. The associations with the other atopic diseases are not surprising due to extensive research around the atopic march. Unfortunately, we are unable to include this analysis in the manuscript due to authorship requirements for the dataset used for the analysis. If the Reviewer determines that this analysis is needed, we would be happy to work through the process with the eMERGE network, but that could take multiple months.

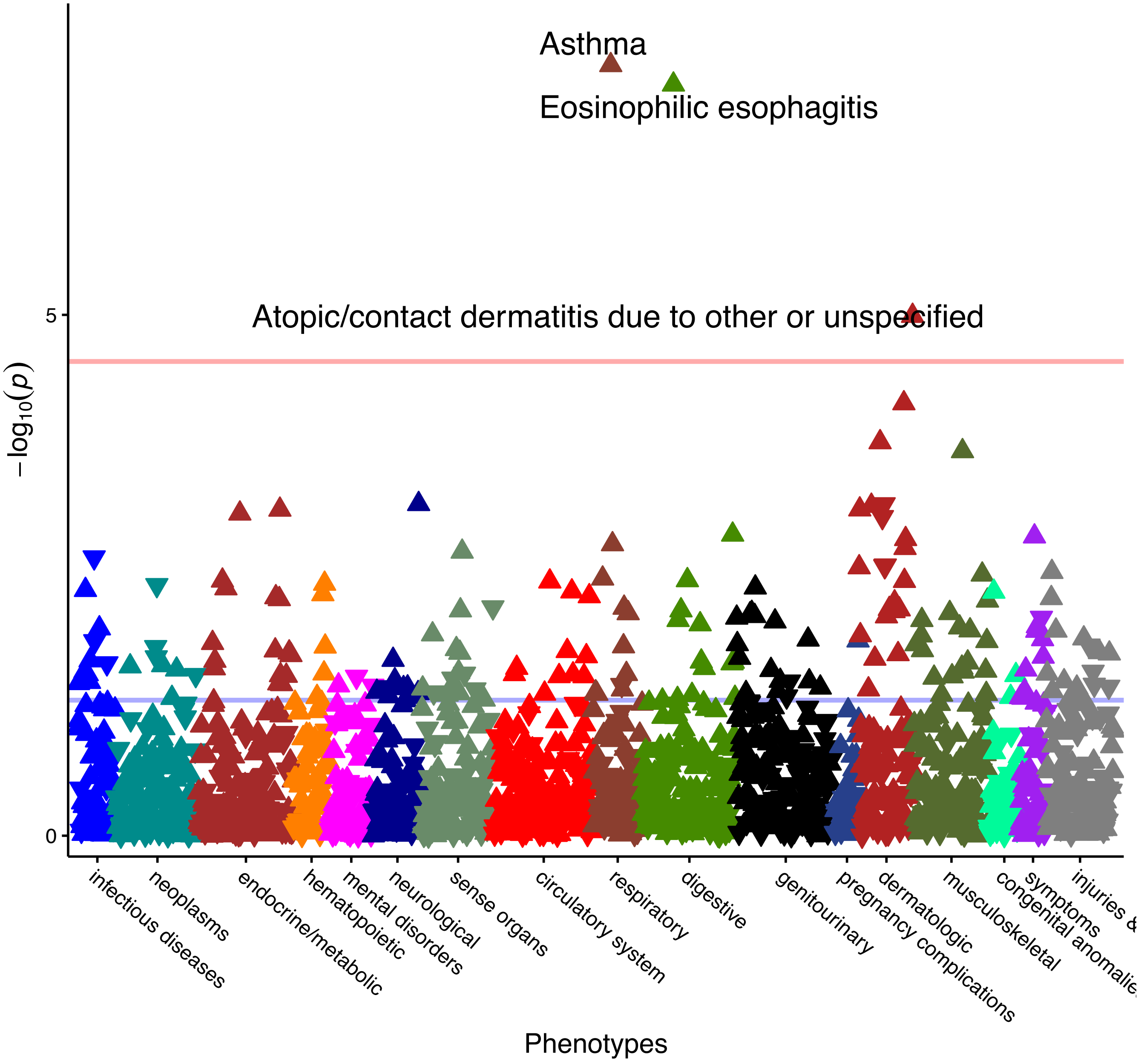

18. The author should make an effort in the discussion, to connect how their data will impact the “atopic march” mentioned in the introduction.

We have added the following section to the Discussion in lines 411-416.

“This study is important for the understanding of atopic dermatitis because a mechanistic understanding of the etiology of the disease could enable efficacious predictive tools and preventative therapeutics. Because atopic dermatitis is the first diagnosis of most patients who find themselves on the atopic march from AD to food allergy to asthma to allergic rhinitis, strategies to prevent and halt AD have the potential to prevent other atopic diseases.”

19. There is an error in the readme file tab in Table 12 that should be changed. See line 15 of the table. S_read mislabeled as weak reads.

This has been fixed.
